# Efficiency aware scheduling techniques in cloud computing: a descriptive literature review

**DOI:** 10.7717/peerj-cs.509

**Published:** 2021-05-04

**Authors:** Muhammad Usman Sana, Zhanli Li

**Affiliations:** School of Computer Science and Technology, Xi’an University of Science and Technology, Xi’an, Shaanxi, China

**Keywords:** Cloud computing, Scheduling algorithm, Scheduling strategies, Scheduling objective, Resource scheduling

## Abstract

In the last decade, cloud computing becomes the most demanding platform to resolve issues and manage requests across the Internet. Cloud computing takes along terrific opportunities to run cost-effective scientific workflows without the requirement of possessing any set-up for customers. It makes available virtually unlimited resources that can be attained, organized, and used as required. Resource scheduling plays a fundamental role in the well-organized allocation of resources to every task in the cloud environment. However along with these gains many challenges are required to be considered to propose an efficient scheduling algorithm. An efficient Scheduling algorithm must enhance the implementation of goals like scheduling cost, load balancing, makespan time, security awareness, energy consumption, reliability, service level agreement maintenance, etc. To achieve the aforementioned goals many state-of-the-art scheduling techniques have been proposed based upon hybrid, heuristic, and meta-heuristic approaches. This work reviewed existing algorithms from the perspective of the scheduling objective and strategies. We conduct a comparative analysis of existing strategies along with the outcomes they provide. We highlight the drawbacks for insight into further research and open challenges. The findings aid researchers by providing a roadmap to propose efficient scheduling algorithms.

## Introduction

In past years, the progression of cloud computing has facilitated the high-speed management of interconnected data centers that are distributed geographically for proposing reliable and extraordinary services (Wu, Lu & Zhu, 2016). The pool of interconnected computers that involves several combined computing resources is referred to as the cloud (Arunarani, Manjula & Sugumaran, 2019). Cloud computing is a system which is built on the different number of distributed servers, cluster, data storage devices, network infrastructures, different software’s and other coherent practical resources which provides the user with all type of highly manageable computing and storage services. Emerging applications and advancements in cloud computing proposed changes in the IT industry and also clarify the vast complications in the improvement of customary IT (Cheng, Li & Wang, 2015).

Cloud computing has grown as an Internet-centered standard model in recent times allowing clients to access according to demands to a united pool of configured resources. Because of this modernization in computing, numerous benefits concerning safety, stack adjusting cost-effectiveness, efficiency, and storage can be perceived. This advancement put forward the computing resources frequently virtualized as facilities for the consumers and hide technical features about resources management (Bittencourt, Madeira & Da Fonseca, 2015).

Scheduling is a decision-making procedure that allows resource distribution between several processes by considering their execution command on the set of accessible resources. The scheduler performs the part of selecting a job in which the computational source will complete each job, thus distributing tasks to perform simultaneously in Cloud distributed system. Accordingly, the execution time of all jobs and the execution time of each job is in accordance with the generated schedule. The scheduler arranges the tasks in resource lines generally following an independent framework. Therefore, in cloud computing scheduling plays a significant role to allocate resources to each task proficiently and efficiently (Bittencourt et al., 2018). In a cloud environment, if the jobs are not organized properly, performance decreases and does not provide the outcomes which are supposed to provide as the cloud processes a vast quantity of data. Hence, efficiency-aware scheduling techniques show a dynamic role in cloud computing (Gupta, Kumar & Jana, 2018).

The task scheduling complications must be controlled at the virtual machine levels because of the virtualization and commercialization of the cloud environment. In the past decade virtualization technology in cloud computing has modernized and commercialized general-purpose computing applications. Through flexible system configuration, server consolidation, elastic resource provisioning and reduction of operation costs benefits are offered in the cloud model. To competently enhance the functioning of cloud, scheduling is a solitary task completed to advance extreme turnover. In distributed systems, the purpose of scheduling algorithms is the distribution of the load on mainframes processors and takes full advantage while reducing the overall task execution time. The most frequently found objective in the literature review of cloud distributed systems is to reduce the execution time which is attained by a suitable allocation of tasks on the suitable resources (Singh, Tyagi & Kumar, 2020).

Internal and external essentials of the resources are set aside and the requirements such as data storage, security, resource expenditures, bandwidth and efficiency regarding time and performance may vary for each job in cloud computing. Security, consistency, and efficiency are the main worries that are noticeable in task scheduling. Henceforth, a secure and efficient scheduling algorithm is mandatory in cloud computing for task and workflow scheduling (Masdari & Zangakani, 2020).

Resource scheduling in a cloud environment becomes a challenging task as the total number of clients using cloud services rises. Resource scheduling under a specified cloud environment presents the process of arranging resources based on certain resource usage rules and regulations among the different cloud users. In resource management, the central technology of cloud computing is resource scheduling. In accordance with the quality of service requirement of the specified applications in resource scheduling, the distribution of appropriate resources to the corresponding virtual machine is very challenging. Although researchers have proposed a lot of resource scheduling algorithms still the cloud service providers find it hard to select a suitable algorithm for their implementations. This is because of the interdependencies, dispersion of assets, heterogeneity of resource types, and uncertainty in the cloud environment Arulkumar & Bhalaji (2020).

In the recent past due to the wide application of cloud computing, there exist a variety of approaches to the scheduling problem. Many researchers (Zhou et al., 2016; Masdari et al., 2017; Arunarani, Manjula & Sugumaran, 2019; Rodriguez & Buyya, 2017; Patil & Aeloor, 2017; Kumar et al., 2019; Kumpati & Pandey, 2021) review the literature and considers the different concerns related to scheduling methodologies, classifies the scheduling challenges and explained existing algorithms from the viewpoint of the application model and resources they consider. Some researchers (Pradhan, Bisoy & Das, 2021; Sandhu & Kumar, 2017) only consider one type of scheduling strategy and provide future challenges and directions related to them. We provide new directives for researchers to establish and classify their work based on the objective they proposed along with providing them with a general idea of existing approaches their limitations and outcomes. We give a broad explanation of the existing literature associated with scheduling techniques and highlighted the future approach recommended in the literature. We analyze the proposed strategies in terms of performance in addition to the scheduling models they adopt and discuss resource scheduling objectives in detail. These benefit researchers in understanding future directions.

Efficiency is analyzed by making assured the scheduling algorithm does what it is proposed to do competently with fewer restrictions and without affecting the cloud computing environment. Efficient scheduling techniques are necessary for the maximum resource utilization in cloud resources with timely workflow execution. We are required to undoubtedly recognize the drawbacks related to various scheduling procedures to overcome these problems and advance toward effective scheduling algorithms.

To be brief, this review has three main intentions:

1. Present new classification for the resource scheduling techniques based on their proposed objectives;
2. Show a detailed analysis of scheduling methodologies their evaluation strategies, outcomes, and drawbacks.
3. Identify relevant future directions to consider which features are to be counted in and which ones to be neglected in the future for efficient scheduling.

## Survey methodology

Merriam-Webster (2018) and Oxford English Dictionary (2018) referred that efficiency is the capability to avoid the misuse of energy, time, money and efforts in giving a required outcome. It is the ability to do tasks effectively while expending as few resources as possible. According to the International Organization for Standardization, efficiency is measured as the resources used up by the user in relation to the completeness and accuracy of goals achieved (ISO/IEC 25010, 2011). In scientific terms “It is a measure of the extent to which input is well used for an intended task (output)” (Sickles & Zelenyuk, 2019).

**Figure 1:**
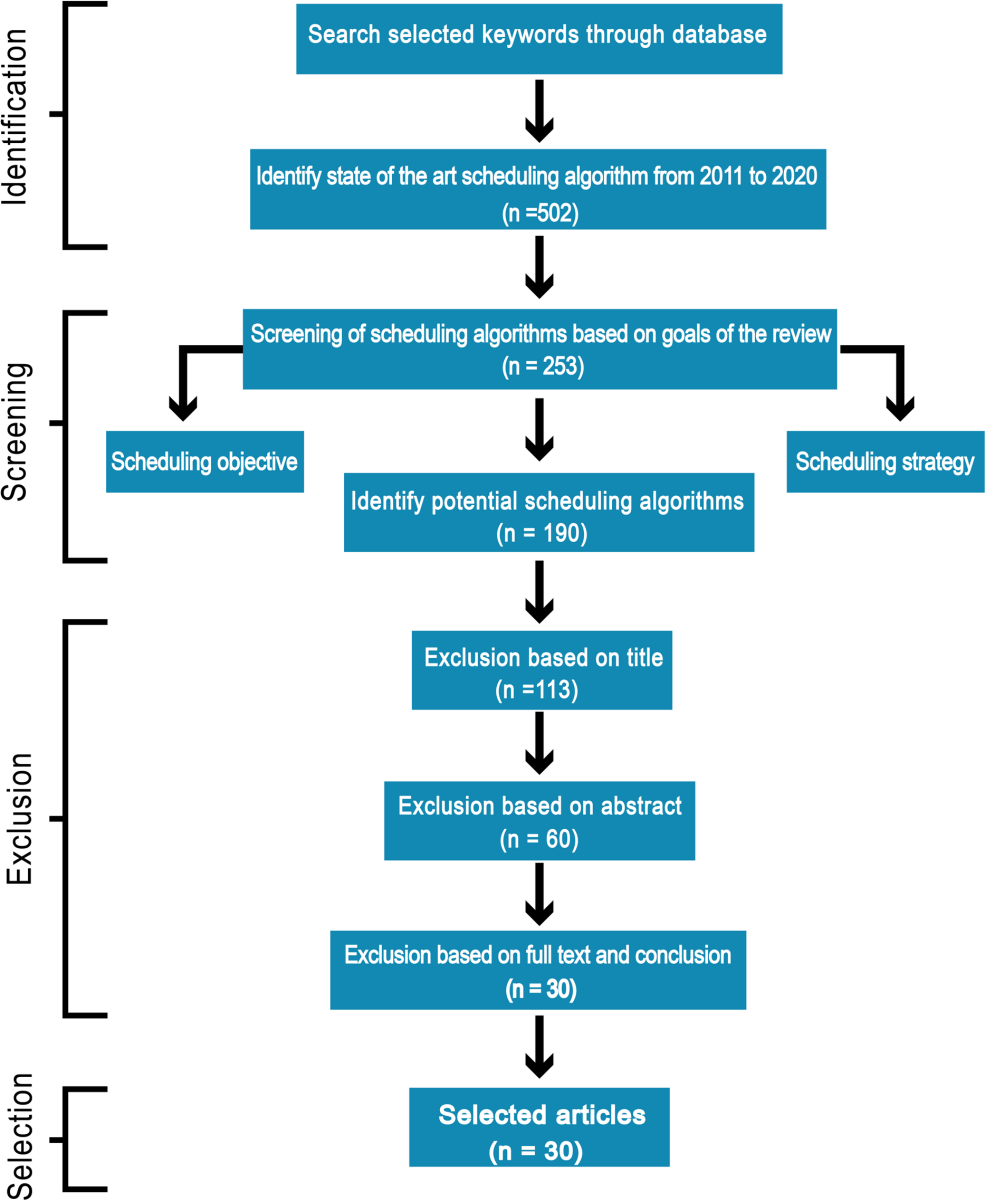
Review technique of articles selection.

**Figure 2:**
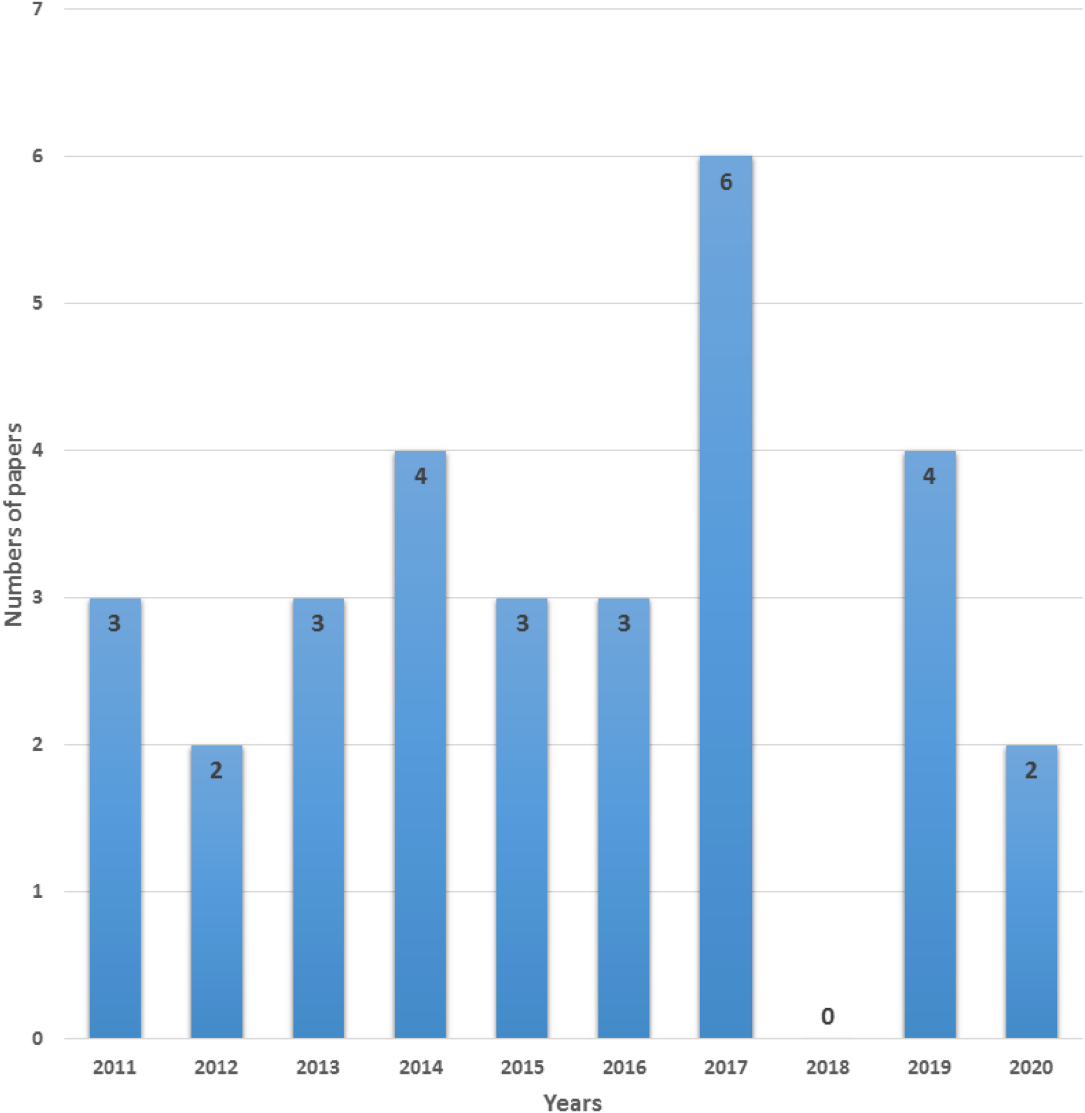
Number of relevant selected papers per year.

To achieve efficiency in performance we suggest seven important goals execution cost, minimum makespan, security, reliability, energy consumption, load balancing and maintaining Service Level Agreement (SLA) that must be considered for an efficient scheduling algorithm. In this paper, we discuss different scheduling algorithms that have become popular in research in the past decade for the cloud computing environment. We carry out an Internet-based search and use terms associated with the objective of this review: “scheduling techniques”, “cost-based scheduling”, “security-based scheduling”, “load balancing scheduling techniques”, “energy-efficient scheduling”, “makespan efficient scheduling”, “SLA based scheduling”, “reliability-based scheduling” and “cloud computing”. Mainly databases such as IEEE explore, Springer, Wiley Interscience, ACM Digital Library, Science Direct, Google Scholar were considered for searching appropriate articles for this review. Most of the identified research papers are from good reputed and high-quality journals and conferences. The review process of the literature is shown in Fig. 1. The number of research papers (n) only published from 2011 to 2020 is focused. According to search terms, more than 500 articles published in top-ranked peer-reviewed journals and conferences were identified, which were scrutinized and reserved 190 significant papers according to the goals of this review. The majority of the research papers were excluded based on the title, abstract, full text, and conclusion of the article because were not in line with our objectives. We distinguished and carefully chosen the 30 most relevant research articles that received a lot of consideration in the industrial and the research area. We carefully investigated all the selected papers year wise as shown in Fig. 2.

This article is systematized as follows; first, we present a detail of the resource scheduling and objectives for efficient scheduling in the cloud. Second, we review different scheduling techniques based on the discussed objectives and show a detailed analysis of scheduling methodologies their evaluation strategies, drawbacks, and future approaches suitable for the cloud computing environment. At last, we provide discussion/open challenges and future research guidelines.

## Resource scheduling

Scheduling is the method to decide, based upon the required parameters which activity should be performed. For the execution of tasks, scheduling is accountable to choose optimal Virtual Machine (VM) through heuristic or meta-heuristic algorithm. Also accountable to observe that quality of service restraint happens. Resource scheduling is of two ways. The demand schedule is the first way in which cloud resources are provided rapidly to the random workload by the cloud service provider. Over-provisioning type of problem can happen because at a single VM there is a chance of executing more tasks, as a result, performance begins to decline. Therefore this method has an issue of uneven distribution of workload. The second way is long-term reservation in which under-provisioning kind of difficultly happens because a large amount of VMs is in an ideal situation.

Excessive wastage of resources and time problems in over-provisioning and under-provisioning increases the cost of services. These types of problems are handled by analyzing and scheduling the upcoming workload in an efficient manner with an efficient resource scheduling algorithm. Scheduling with resource provisioning is required for providing the VMs to users to fulfill the user’s requirements without violation of SLA. Resource provisioning based on upcoming workload helps us at the starting to recognize the requirement and expectation of the cloud users. After workload is appropriately observed SLA agreement is defined among service providers and users. The fitness function is calculated for each workload based upon the necessary quality of service (QoS) parameters or it is matched with the value evaluated without considering QoS parameters. If resource provisioning is ended effectively then with the help of the scheduler tasks are process in the identified deadline and budget scheduling algorithm. Running resources of the cloud are checked and each resource load is measured, before allocation of tasks or workload at the VM (resources). If any VM is in beyond utilized stage task is not assigned to resources. Check the state that running VM is sufficient or not executing the workload, additional upcoming workload is the plan with the existing resources. The necessary QoS parameters are calculated if running resources are not sufficient then using the concept of horizontal scalability and the resources are enhanced Singh & Chana (2016a).

Centered upon the idea of resource scheduling in cloud environment we have reviewed various research papers mostly associated with scheduling (resource allocation, task scheduling, workflow scheduling, etc.) techniques. Resource allocation and task scheduling allow providers to enhance the utilization of resources and profits up to their limits. In cloud computing resources, in terms of the performance allocation of resources and scheduling are the main hurdles. To properly utilize the available resources the process of organizing the tasks (incoming request) in a certain manner is task scheduling. Workflow scheduling describes as the set of interrelated tasks and for better resource scheduling their allocation amongst different accessible resources Gawali & Shinde (2018).

Many kinds of scheduling mechanisms exist for example static and dynamic scheduling, preemptive and non-preemptive scheduling algorithm, etc. Static scheduling algorithms require the data concerning resource (node processing capacity, memory, processing power, etc.) and task (number of tasks, length of the task, and deadline of tasks) in advance. When the difference in workload is small and system behavior is not changing repeatedly static algorithms work well. In a cloud environment load fluctuates suddenly, hence static algorithms are not an appropriate option. QoS parameters didn’t optimize by static algorithms. Static algorithms are very easy to implement, but in the real environment don’t deliver worthy results. In dynamic algorithm data regarding the node and task is not required in advance but to monitor the node it is required continuously. For cloud environments, these algorithms are further accurate and efficient because they relocate the task to underloaded node from an overloaded node if some node is in an overloaded state i.e. with load alterations (decrease or increase) at a node algorithm condition alters frequently. In a preemptive scheduling algorithm, tasks can be migrated to other resources and can be interrupted at the existing execution. In a non-preemptive scheduling algorithm, task will not be free when a task is assigned to a resource in cloud until the task is finished. A task without being delayed or interrupted is completely executed at the resource. In cloud computing at one resource, one task is executed i.e. during the execution of tasks interruption is not acceptable Singh & Chana (2016b).

## Objectives for efficient resource scheduling

**Figure 3:**
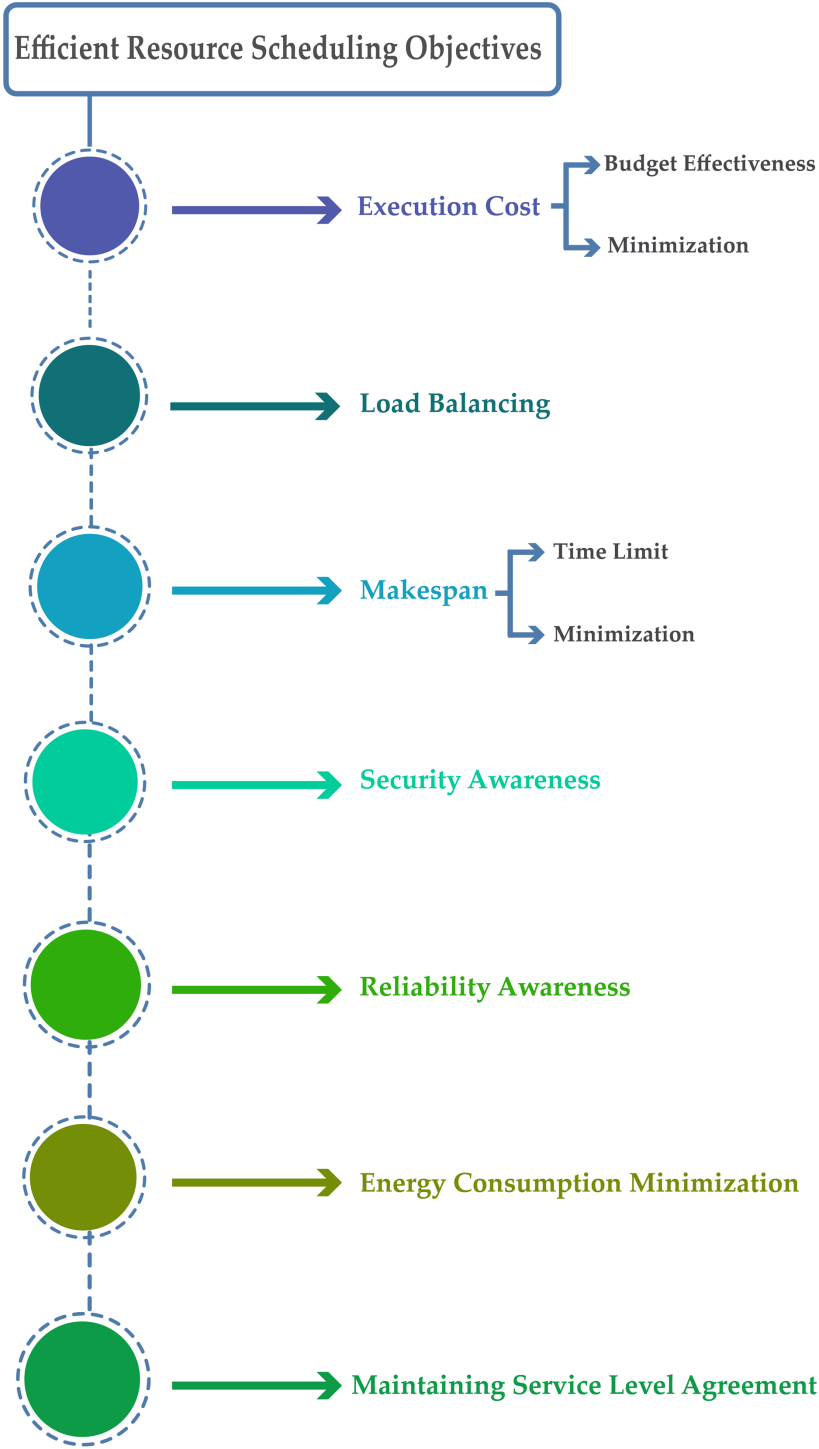
Efficient resources scheduling objectives.

Attain the projected aim through transmitting tasks to suitable resources for implementation is the basic goal of efficient scheduling. Presently, the objectives for efficient scheduling in cloud computing include execution cost (budget-effectiveness), reliability, minimum makespan, security, load balancing, minimum energy consumption, and maintaining service level agreement. According to the analysis, the foremost objective for efficient resource scheduling is shown in Fig. 3.

### Execution cost

The main challenging problem in clouds is to execute tasks while minimizing the cost along with execution time acquired by consuming a set of heterogeneous resources over the cloud at the same time. Algorithms that are considered for the cloud environment need to be budget effective for the setup. If they are ineffective to do so, then the cost of leasing VMs, transmitting data and consuming the cloud storage may be noticeably high. Algorithms can balance the cost by fixing or minimizing the amount of cost spent on the resources. The total cost experienced by task execution comprises several cost components such as data transfer cost and estimated cost of cloud setup. The cloud consumer requires paying reasonable managing costs to the cloud supplier. Most algorithms balancing cost with other important objectives related to performance for example security and energy consumption. The work associated with cost minimization was studied for the purpose of this research and observe helpful measures by which reducing cost for the cloud architecture.

**Figure 4:**
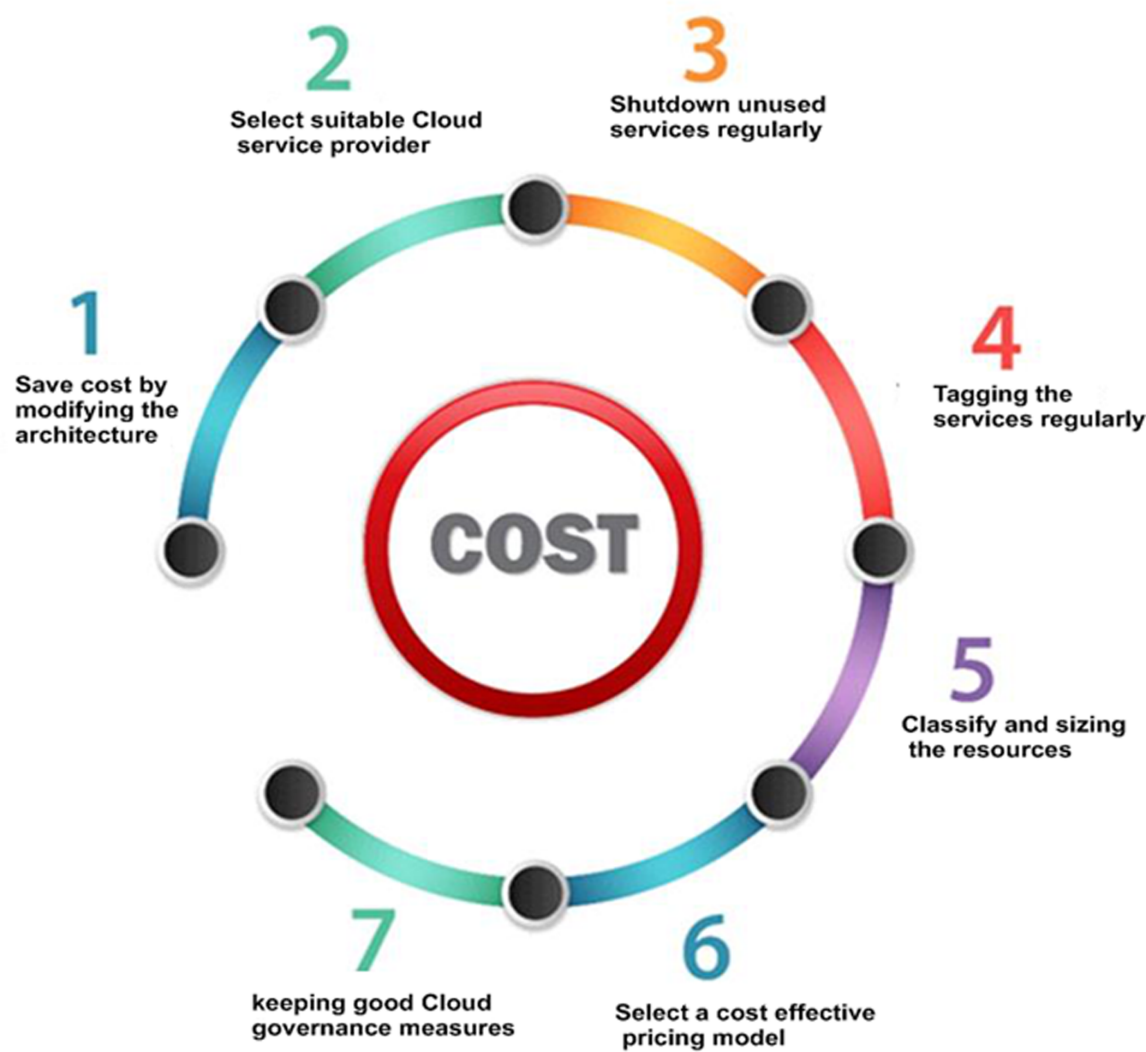
Measures to control overall cost in cloud.

Figure 4 explains the basic strategies to control the overall cost in the cloud. Effective cost management starts with a detailed analysis of the entire set-up. Inadequate tuning of the architecture of the cloud can lead to poor management of the resources. To escape over-utilization of resources dynamic resource allocation can benefit in balancing loads correctly. Inadequate management of cloud budgets and systematizing tasks such as code deployment, security and compliance, settings and configurations, backup and storage, is a prominent cause of overspending and under-utilizing resources. Underutilized resources must be removed and avoid excessive waste by sizing instances so that the process of deploying instances is not only becoming faster also make sure that only specific resources are provisioned. Must choose the right cloud service provider (CSP) and have broad visibility on the cloud services used, recognize the actual usage pattern. Make sure that the cloud instances you decide on with CSP are exact suitable for your organization’s requirements. Another important strategy to save cost is tagging of services that help in many purposes. This provides organizations better visibility across functions into cloud cost and usage. Lack of appropriate tags may possibly end up paying extravagantly for the services. A tag management strategy in cloud can increase the efficiency of operations management and prevent mistakes. The overarching goal in cloud computing is to attain excellent performance at the lowermost possible costs by keeping network settings and storage optimal. Ballooning costs in cloud occurs if you are failing to re-size or right-size your cloud resources regularly. It is very difficult to achieve balance in maximizing the workload without overspending. Regularly perform an investigation of your needs and implement appropriate re-sizing accordingly. If you aren’t scheduling your resources it is necessary to shut down unused services by limiting the data and actions to control costs periodically. According to the work hours and workloads, the starting and stopping of schedules can be configured. There’s no purpose to keep them active if resources are not used by anybody and pay for them.

The alignment of cost-effective plan ensures the expected benefit in cloud and provide a good balance between cost and performance. In addition to the strategies mention above, good governance is also required in setting policies for the cloud usage, making appropriate budgets and in overall cloud cost management.

### Load balancing

**Figure 5:**
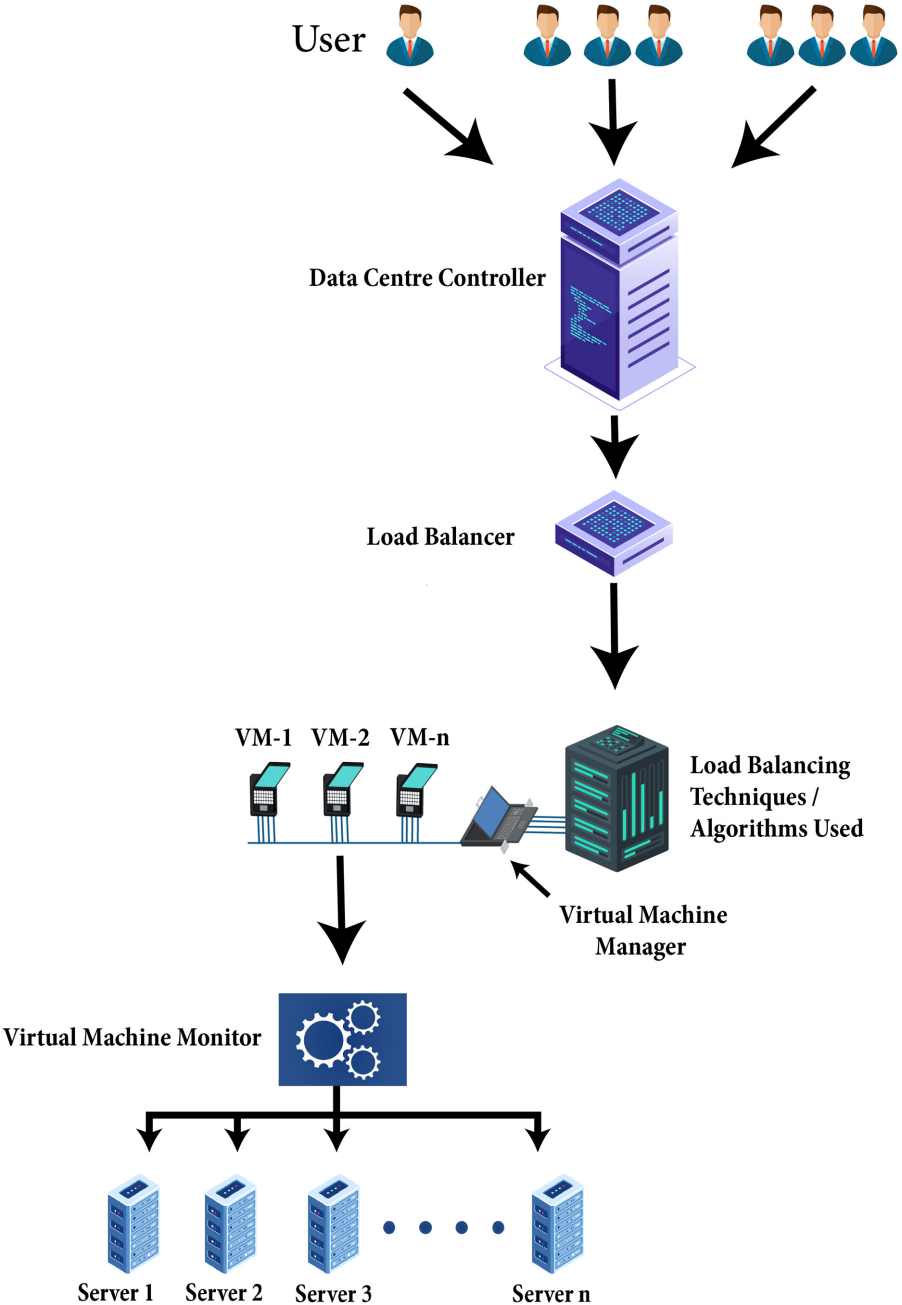
Technique of load balancing algorithm.

Particularly in a cloud environment load balancing is vital within extensive data handling applications. As soon as in cloud environment scheduling algorithm is planned to schedule jobs, a scheduler uses the load balancing techniques amongst cluster nodes into a concern to boost the use of resources and to escape the chances of overloading resources in the cloud. Load balancing is a method that allocates the excess dynamic local workload uniformly across all the nodes. Load balancing algorithms are used for achieving resource utilization, better service provisioning, and enhancing the total performance of the system. Figure 5 explains how the load balancing algorithm works. The client’s incoming network traffic efficiently distributed across multiple servers at the backend. Requests generated by clients or users route towards data centers. The data center controller sent the requests to the load balancer on the potential server where an effective load balancing algorithm distributes the network load or client requests with efficiency on VMs. Virtual machine manager provides the VMs the requests of clients or load of a network to execute properly. Virtual machine monitor maintained the physical resources on which VMs are created. However, the load of network and user requests assigned to VMs shows the collective performance of the VMs.

Load balancing algorithms help schedule and allocating tasks to suitable VMs according to their requirements to distribute the cloud workload effectively. Checks the status of VM and details of resources. The VMs status can be determined with respect to a threshold as overloaded, under-loaded, or balanced. To schedule tasks for execution, the resources are allocated. The efficacy of the allocation policy and the scheduling algorithm determined the strength of the load balancing algorithm. Henceforth, load balancing methodologies provide many benefits like system stability, increases resource utilization, build fault tolerance system, reduce execution time, speed up response time, etc. with a particular cost and within a definite period.

### Makespan

Makespan is the period between the onset time of the leading job and the closure time of the last job. In short, it is generally calculated by the completion time of every job and the modeling cost among them and that is the deadline for the completion of the workflow schedule. In cloud computing tasks scheduling with the lowest makespan and less waiting time is considered as an optimization problem. The important concern from the end-user perspective is to keep the makespan and response time minimum while maintaining the QoS. However, the main concern of the CSPs is revenue generation by the maximum valuable utilization of the existing resources. Considering the concerns of end-user and CSPs it is required to suggest and implement scheduling techniques that are capable of managing maximum resource utilization of the accessible pool of resources efficiently and scheduling requests of large numbers.

To define the task scheduling problem it is required to observe both resources of VM utilization and makespan. How well the resources are used in the cloud is defined as the utilization of VM. Generally, the Utilization rate is inversely proposal to Makespan. The efficient scheduling algorithm must schedule the tasks in such a technique that VM utilization is maximum and makespan is minimum. In most of the resource scheduling approaches, the time limit of a workflow is a leading purpose. We study and assess algorithms concerned with the minimization of the total time takes to execute the complete workflow.

### Security awareness

**Figure 6:**
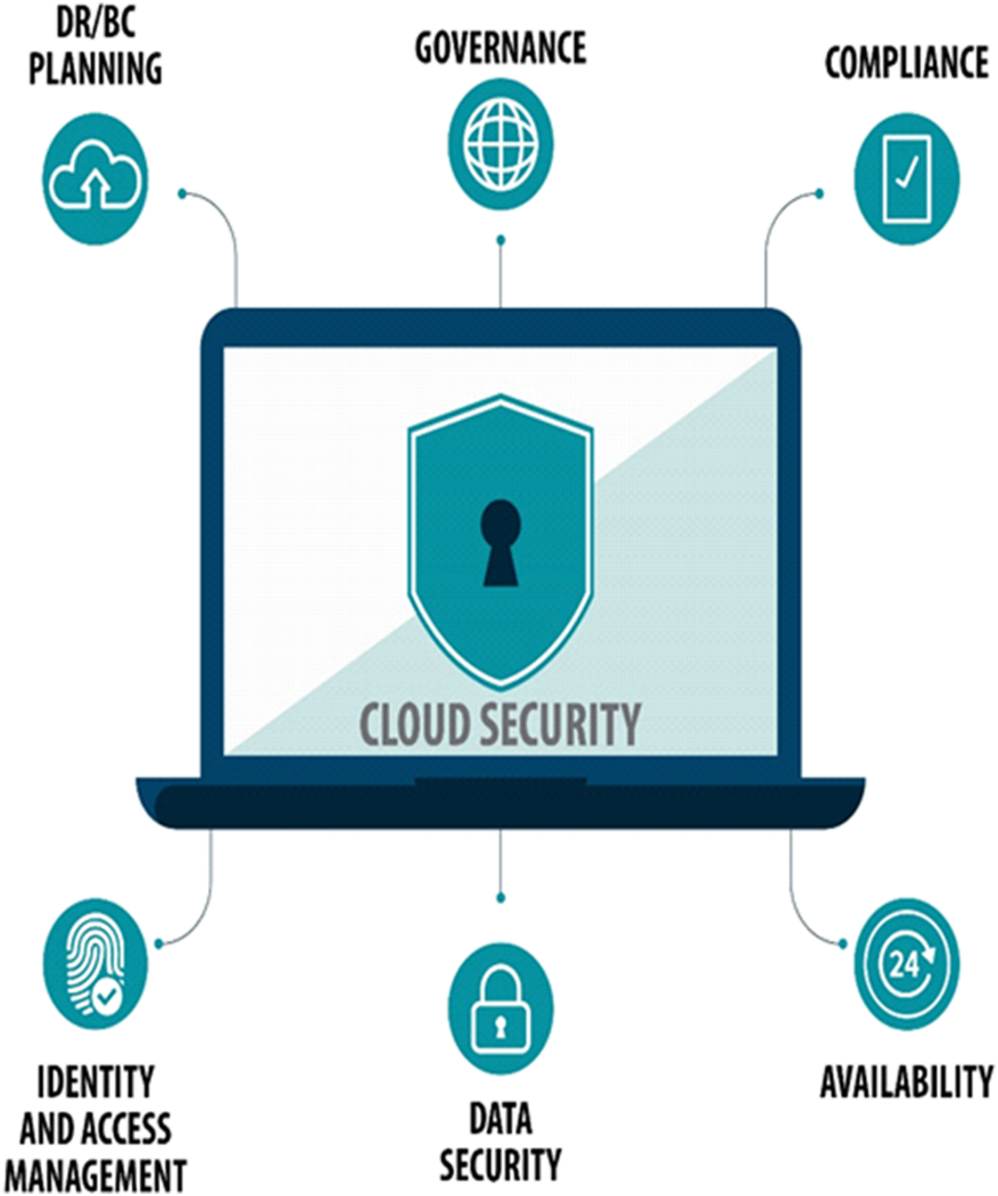
Measures for secure cloud deployment.

Data privacy and security come to be a dynamic subject when an organization chooses to use a cloud environment. Figure 6 shows that in enabling secure cloud deployment following measures are required in the cloud architecture. To put customers’ minds at ease, cloud service providers must ensure that regulatory compliance is required, the cloud environment and the data must be secured, up-to-the-minute insight is required into the existing state of security, timely warnings of out-of-bounds circumstances, recognize the root cause of problems and remediate them. Good governance is required to make sure that only authorized personnel have access to the system and equipment. Must have disaster recovery/business continuity (DR/BC) planning. Data is stored with a third-party provider so control over the data and visibility is very limited. Therefore this raises the question of how it would be precisely secured. Cloud security is the set of practices and strategies for guarding data and applications that are held in the cloud. Cloud security is very extensive, and it is certainly not possible to avoid every variety of attacks but a well-designed cloud security approach massively reduces the risk of attacks.

Scheduling associated security challenges are considered to be the main issue in cloud computing. Cloud setup is more prone to scheduling and network-related attacks. Furthermore, cloud operations are highly dependent and closely coupled with scheduling methodologies. As organizations and people migrate their data into clouds the ratio of threat and attacks vividly increases. In the time of need, data must be correctly encrypted, protected, controlled and transmitted. Data loss and leakage, data recovery and location, data redundancy and availability, data privacy and protection have been deemed as important and major issues. The possible overlooked security configurations and lack of appropriate installations of network firewalls within cloud networks make it easier for unauthorized access to the cloud for hackers. unauthorized access is a threat to confidentiality, integrity, and availability of services and data. For technical issues and fate sharing incidents it is extremely important to detect and fix the errors and faults transfer from a corrupted server to each VM and to avoid them from reoccurring it is required to implement the best practices. The security misconfiguration may possibly occur in the framework, application stack, platform, in custom code with a web server. In the cloud, servers are responsible for various services such as data storage and directory service and are considered as the mainstay of cloud infrastructure. Another important issue is the reliability of suppliers to control hardware and data access a good check on the staff is required. Through virtualization, cloud serves numerous real-time users, so different users share the same hardware and software resources. Data leakage from one server mate to another may happen due to the multi-tenancy ability of the cloud. Attacks may also occur such as VM-to-VM so there should be no window for security reasons and because compromised VM is becoming the suitable center for future attacks.

With increasing concerns regarding information security in the cloud, computing awareness is also developing regarding the usage of security algorithms in processes and data systems. Several technical applications may be needed so that the internal and external data are controlled in a protected way. We analyze how algorithms handled these security problems and how the provider presented different security services. Algorithms control data firmly and save the data in a proper manner that resources having an advanced level of security are utilized to store or execute them.

### Reliability awareness

**Figure 7:**
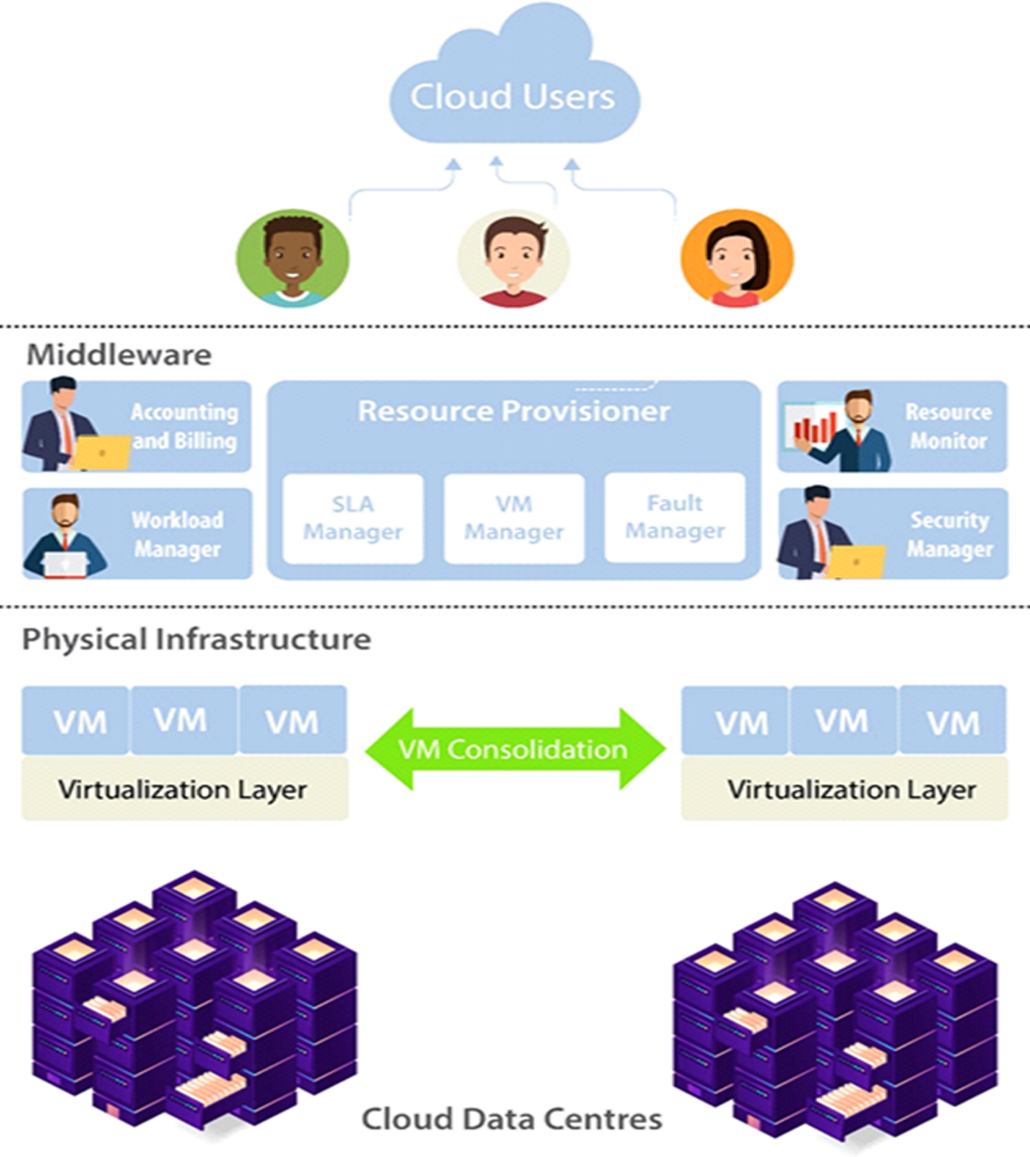
Model for reliable cloud computing services.

Distinctly from the vital objectives like budget criteria and response time, workflow reliability is also very necessary to consider. In Fig. 7 model for reliable cloud computing services is presented, which offers effective control of cloud resources. The three important layers of this model are Cloud Users, Middleware and Physical Infrastructure. Cloud user submits their requirements and in terms of SLA outlines necessary services. To handle the incoming consumer workloads workload manager is deployed. For resource provisioning transfer the workload to the middleware. Middleware includes five subcomponents, is the main layer of the model includes accounting and billing, resource provisioner, workload manager, security manager and resource monitor. Physical Infrastructure contains cloud data centers that are used to execute workloads of the cloud. VM migration or consolidation is implemented for execution according to the VM manager policy. System reliability is considered as the possibility that a system is operational without any failures in a time interval. High availability and reliability have always been the most important area of interest in distributed systems. In cloud computing provided that extremely reliable services are necessary for keeping customer satisfaction, confidence, and avoiding revenue losses. There are chances that the cloud job can be concluded positively within the user’s quality of service limits even if resource failures take place. This happens when certain common tactics for example backup/restart techniques and active replication possibly applied in the cloud scheduling algorithms. On the other hand, algorithms must be aware of the extra costs related to replication of tasks for example storage of data. Although several techniques have been suggested for cloud reliability and availability. However, there are no broad studies that entirely cover different aspects of reliability in the cloud for example the connection between reliability, availability, and maintainability while avoiding performance degradation how to keep the reliability and high availability. Hence, must consider the provider’s requirement for having highly utilized systems with reliability and availability.

### Energy consumption

Cloud computing encourages all the researchers to create an effective environment of the cloud for its customers and more cost-effective for the providers as its demand is growing progressively. Datacenter which includes the collection of servers on which applications run and information is stored is the most prominent in cloud computing. Data center consumes a lot of power, the optimization of energy utilization is one of the main challenges faced in cloud computing. Large numbers of data centers are being built to satisfy customers’ requirements. On the other hand, the data centers put away large quantities of energy that appeal negative attention. For that reason, sources that provide cloud are critically pressurized to decrease their energy consumption. Only a small number of algorithms have been developed in recent times that consider to cover challenging scheduling issues as they attempt to find interchange between the cost and energy consumption performance. There are few techniques used to decrease the energy consumption in a cloud environment for example Virtual Machine (VM), Dynamic Voltage and Frequency Scaling (DVFS), Migration, and VM Consolidation. VMs levels and the absence of knowledge and control of the physical infrastructure restrict the abilities and added more complications in this matter.

### Maintaining service level agreement

**Figure 8:**
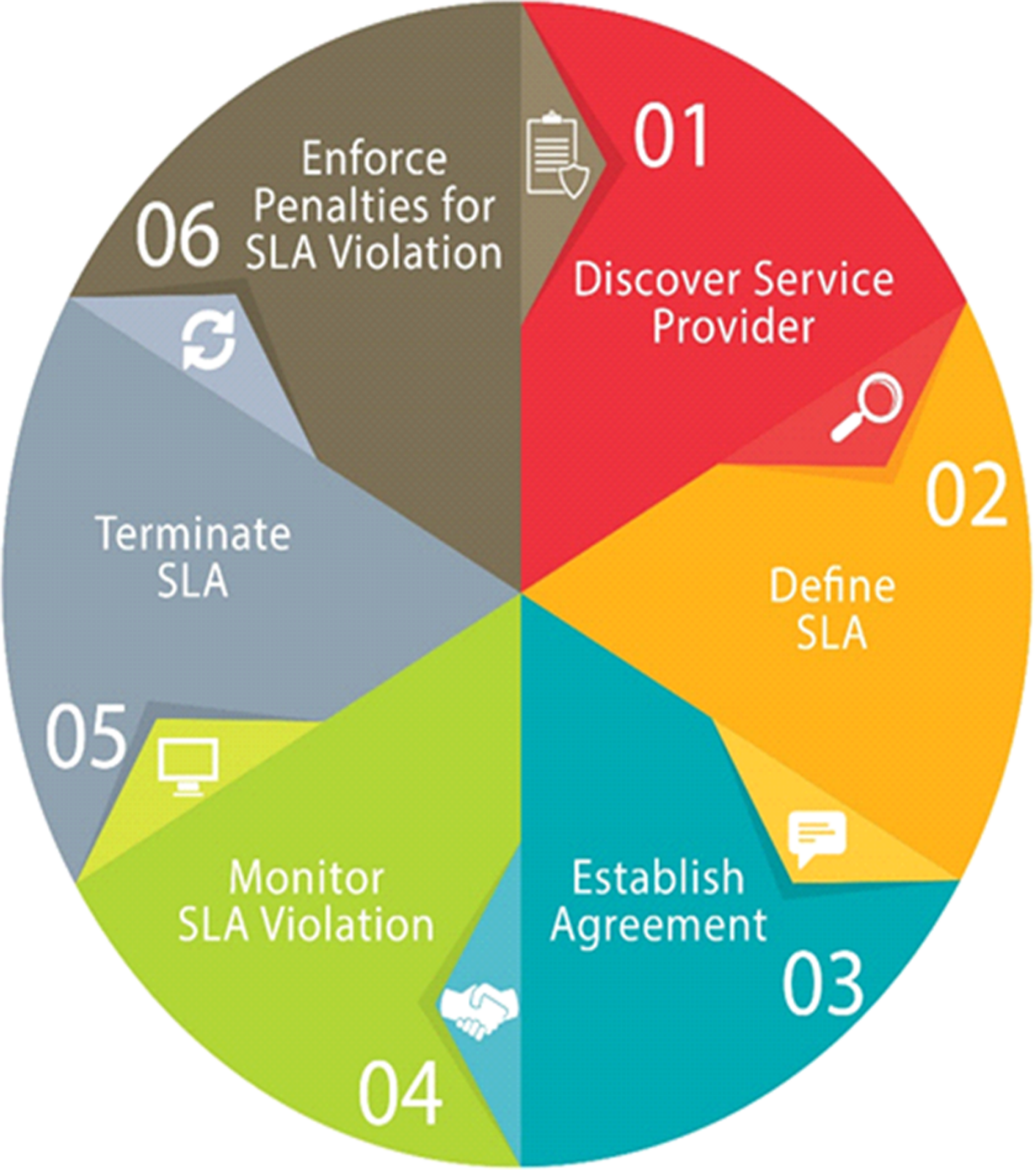
Steps for maintaining Service Level Agreement.

SLAs get more and more prominence in the area of cloud computing. SLA is an agreement between a client and a CSP for performance negotiation in which the CSP gives assurance of the functional and non-functional quality of service parameters. Subsequently CSPs penalties for violating SLAs as well as have to pay for the hardware used, they are enthusiastic to accomplish these agreements while simultaneously improving the utilization of their resources as shown in Fig. 8.

In cloud computing, SLA is the main concern because it describes essential parameters such as the pricing, period of service, security, uptime, and downtime. Despite that, from one CSP to another the service possibly differs. In the heterogeneous multi-cloud environment the association of the CSPs is very daring and challenging, and it is not well described in the current literature. Formerly, in the cloud, all SLAs were discussed between a consumer and the service provider. Currently, most SLAs are standardized until a client turns out to be a giant consumer of cloud services with the beginning of huge utility-like cloud providers.

## Resource scheduling techniques

**Figure 9:**
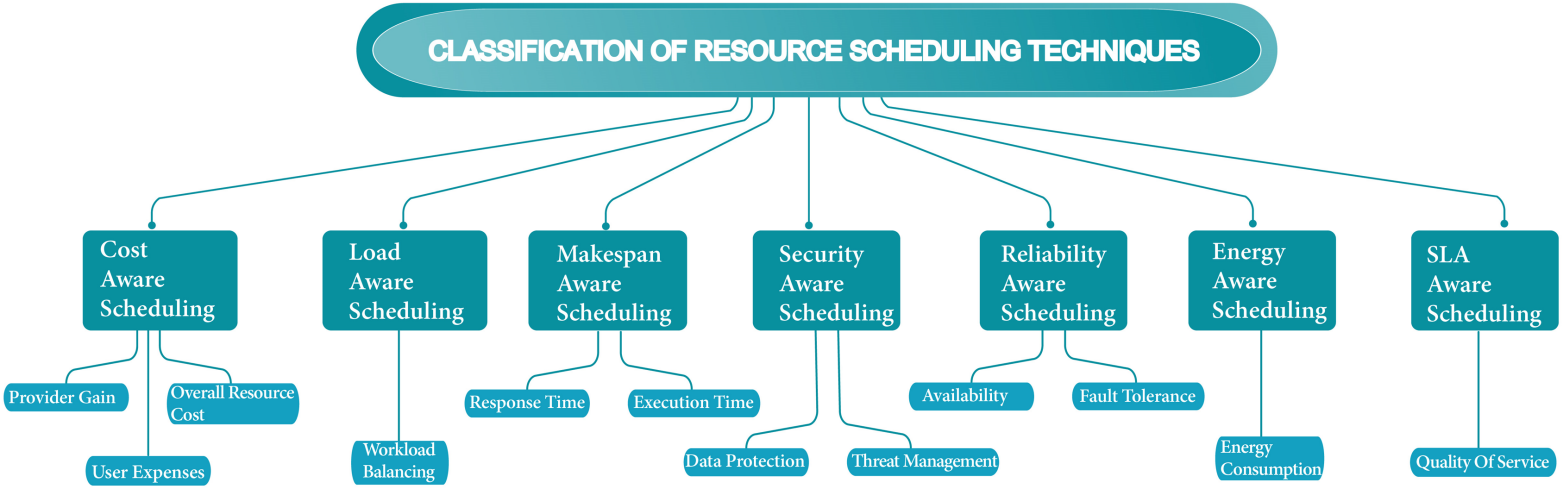
Classification of resource scheduling techniques.

Over the past few years, scheduling techniques have become technologically innovative concerning the growing cloud computing environment. More advancement regarding cloud resources and workflows techniques has become essential in the scheduling process. Scheduling techniques have become a firm process with a growing number of jobs and users. In the cloud, most scheduling methods only focus on either computation-intensive or data-intensive job types. Focused on one job type in the schedule is not suitable according to the perspective of all environments, and occasionally on the other side leads to wasting of resources. In this analysis, we search numerous cloud scheduling algorithms interrelated with the objectives which are required for efficient resource scheduling. Figure 9 shows the classification of resource scheduling techniques. Table 1 shows the summarization of scheduling techniques based on their scheduling strategies, objectives, evaluation strategies, outcomes, and drawbacks. Table 2 shows the summarization of the future approach of scheduling techniques.

**Table 1:** Summarization of scheduling techniques based on different aspects.

Algorithm reference	Scheduling strategy	Scheduling objective	Evaluation strategy	Outcomes	Drawbacks
Byun et al. (2011b)	Heuristic	Deadline and cost	Real cloud	Estimate resource capacity per time Minimize the resource cost while meeting the deadlines	Not perform well if task on critical path delayed too much or if some tasks crash in worst case. Load/store bandwidth and data intensive application can be an issue because PBTS does not consider Input/Output contention when determining the start time of task.
Byun et al. (2011a).	Heuristic	Deadline and cost	Real cloud	To execute the workflow minimize the resource capacity within a given deadlines	In determining the start time of the task not consider network contention Still, the execution time may increase if lots of tasks use the network at the same time. Less efficient as compared with a fully elastic resource allocation system
Bittencourt & Madeira (2011).	Heuristic	Cost	Simulation	Reduced the cost with desired execution time	Unable to deal with multiple workflows
Tan et al. (2013).	Service-oriented	Cost and deadlines	Simulation	Satisfy the deadline constraint by reducing the execution cost significantly	In response to changes in the cloud environment not suitable for adoptive collaborative model. More extension is required Context-aware collaboration systems model is necessary to support an unreliable and uncertain cloud environment
Malawski et al. (2012).	Hybrid HO	Cost and deadline	Real cloud	In a given budget and deadline constraints maximize the no of user prioritized completed workflows	Data storage and transfer cost are not considered.
Rodriguez & Buyya (2014)	Meta-heuristic	Cost and deadline	CloudSim simulator	Decrease the total workflow execution cost while satisfying deadline constraints	Extension of algorithm is required to include heuristics that ensure a task is allocated to a VM with enough memory to execute it. Not consider data transfer cost
Mansouri & Javidi (2019)	Hybrid heuristic	Cost, makespan	CloudSim Simulation	Decrease makespan, increase processor utilization and decrease the response time of computing -intensive and data-intensive jobs.	Additional investigation is required to understand the interrelationships between the mentioned parameters such as processor utilization, makespan, degree of imbalance, bandwidth consumption etc. Further improvement is required in case of business cloud to investigate the security aspect.
Chen et al. (2015)	Heuristic, Task clustering	Load balancing	Workflow sim simulator	Compared to baseline execution decrease the run time of applications	Not suitable if workflows have no explicit pipelines
Kumar, Gupta & Jana (2016)	Heuristic	Load balancing and makespan	Real cloud	Maximize average cloud utilization and minimize the makespan	The cost is increased. Only favorable when using forward load
Casas et al. (2017)	Exponential graph-based	Execution time, System utilization and Monetary cost	Real cloud	To balance system utilization splits scientific workflows into numerous sub workflows via parallelization	Not worked with diverse workflow dependency patterns. Overhead time is large
Kashikolaei et al. (2020)	Meta-heuristic	Load balancing, makespan, and stability	Simulation	Improve the low CPU time, load balancing, and makespan, Enhance stability in cloud timing	Not considered deadlines and security of data
Panda & Jana (2019)	Probability theory	Load balancing	Simulation	Extraordinarily balance the load of the virtual machines	The relationship between performance and the network load is not considered. Execution costs, deadlines and the random arrival of tasks are not considered.
Niu, Ong & Nee (2012)	Meta-heuristic	Makespan	Simulation	Solving job-shop scheduling problem	Computational time become longer when the complexity of instances increases
Zhu et al. (2015)	Meta-heuristic	Makespan and cost	Real cloud	Minimize cost and makespan simultaneously	Not considered monetary cost and time overheads of storage and communications
Poola et al. (2014)	Heuristic	Makespan and cost	Simulation	Provide a fault-tolerant and robust schedule. Minimize total cost and elapsed time	Less reliable. Experiments conducted with a 10 percent failure probability
Raju & Devarakonda (2019)	Heuristic	Makespan	Cloud sim simulator	Decreasing makespan together with load balancing ability Reducing cloud structure cost.	Not suitable if more number of cluster are formed in small period
Zhou et al. (2019)	Heuristic	Makespan and monetary cost	Simulation	Reduce execution cost. Achieve better cost-makespan trade-off	Assuming fixed task execution time. Not considered dynamic voltage and frequency scaling DVFS technique
Zeng, Veeravalli & Li (2015)	Heuristic	Security, budget, and makespan	Real cloud	Shorter makespan with secured scheduling under budget constraint	Priority is not considered.
Li et al. (2016)	Meta-heuristic optimization PSO based	Security and cost	Cloud sim simulator	Overall workflow execution cost is reduced while satisfying the risk constraints optimization and deadlines	Priority, load balancing is not considered.
Arunarani, Manjula & Sugumaran (2017)	Hybrid optimization	Security and cost	Cloud sim simulator	Overall execution cost is minimized while satisfying the risk rate and deadlines constraints.	Bandwidth and load balancing is not considered
Poola, Ramamohanarao & Buyya (2016)	Heuristic	Cost, fault tolerance and Execution time	Cloud sim simulator	Provide fault tolerance robust schedules with minimum cost and makespan	Not considered multiple data center and data transfer cost
Rehani & Garg (2017)	Monte Carlo method	Reliability and makespan	Cloud sim simulator	High reliability of task execution with the fewer possibility of occurrence of any failure. Increase overall makespan of the application	Not considered Budget, deadline, and security
Xie et al. (2017)	Enough replication, Heuristic replication	Cost and reliability	Simulation	Satisfy applications reliability requirements. Low time complexity and minimize redundancy	Resource usage and schedule length minimization is not considered.
Swain, Saini & Sahu (2020)	Heuristic	Reliability and execution time	Simulation	Reduce the weighted estimated failure probability	Not considered the cost. Not suitable if more than one number of VMs required for a task
Duan et al. (2017)	Prediction model	Energy	Cloud sim simulator	Reduce consumption of energy and system resources	Each request occupies a single VM in this model. This approach leads to under-utilization of resources
Pietri et al. (2013)	Static	Energy, budget, deadline and workload	Cloud sim simulator	Without compromising the no of workflows completed in an ensemble minimize energy consumption	Delayed provisioning. Data transfer cost is not considered
Yassa et al. (2013)	PSO based heuristic	Energy, cost, and makespan	Simulation on real-world and synthetic scientific applications	To minimize energy consumption	Different levels of voltage supply workloads are used. These multiple voltage involves a compromise between the energy and quality of schedules.
García, Espert & García (2014)	Dynamic	Cost, efficiency and SLA	Simulation considers some realistic workload profiles	Minimize cost and provide maximum efficiency under heterogeneous utilization pattern	Not considered disaster recovery protocol and distributed SLA monitoring
Garg et al. (2014)	Static	Resource utilization and SLA	Cloud sim simulation	QoS requirements of the customer are met as stated in SLA. Maximize resource utilization.	Not considered cost. Not reliable for workflows and parallel applications.
Panda & Jana (2017)	Single and double phase	Makespan, cost, and SLA	Simulation	The stability between the gain cost and makespan of the services acheived	Parameters like performance, availability and reliability are not considered

**Table 2:** Summarization of future approach of scheduling techniques.

Algorithm reference	Future approach
Byun et al. (2011b)	How to handle load/store bandwidth issue when workflow applications are data-intensive?
Byun et al. (2011a).	Change the resource capacity according to the status of resources and workflow dynamically.
Bittencourt & Madeira (2011).	Considering the potential presence of external load in private resources could improve the scheduling decisions. Estimate the existing bandwidth in between two public clouds to decide when the dependent task can be put in different public clouds
Tan et al. (2013).	Determine how dynamic runtime changes could affect the statically predetermined schedule. In response to changes in cloud computing environments plan to modify the TWFS algorithm for adaptive collaborative model.
Malawski et al. (2012).	Investigate utilization based autoscaling with new accurate and advance workflow schemes build on feedback control. As various data storage options available on clouds plan to extend the infrastructure and application model. To study heterogeneous environments including private and community clouds that comprise of multiple cloud providers and VM types.
Rodriguez & Buyya (2014)	Extend the resource framework and observe the data transfer cost among data centers to deployed VMs on different regions. To include heuristics modifying the algorithm that confirm a task is allocated to a VM with enough memory to execute it. For deploying applications in real life environments aim to implement this methodology in a workflow engine.
Mansouri & Javidi (2019)	For business clouds, investigate the security aspect. Use different optimization approaches such as genetic algorithm and ant colony optimization to determine the performance of the scheduling approach in given experimental setups.
Chen et al. (2015)	Analyze more workflows applications using asymmetric structures to study the connection between metric values and workflow structures. Formation of a portfolio clustering algorithm, which selects several clustering algorithms, and according to the dynamic load, dynamically chooses the most appropriate one.
Kumar, Gupta & Jana (2016)	Adopt dynamic load balancing in the cloud. Considering regular load balancing and task migration over the virtualized setup from one VM to other.
Casas et al. (2017)	How to significantly reduce scheduling overhead time?
Kashikolaei et al. (2020)	For scheduling and load balancing in a dynamic environment combination of collective intelligence algorithms and evolutionary algorithms based on several populations are required.
Panda & Jana (2019)	Consider the random arrival of tasks with execution costs and deadlines. Study results by using real cloud environment for the proposed algorithm.
Niu, Ong & Nee (2012)	Apply IWD to different scheduling complications like flow-shop scheduling and open-shop scheduling. Modified IWD algorithm to increase the diversity of the solution quality as well as the solution space.
Zhu et al. (2015)	In a single schedule, consider how to use instance type groups, more than one pricing schemes, or even multi-Clouds.
Poola et al. (2014)	Consider different cost models with existing system offered by the cloud and their effect on the workflow robustness.
Raju & Devarakonda (2019)	Improving existing technique to decrease more utility to additional assigned machines and in a shorter span more number of cluster formations.
Zhou et al. (2019)	In hybrid clouds when solving the workflow scheduling issues consider a variation of execution time of tasks and also effects of DVFS on reliability, cost, and makespan.
Zeng, Veeravalli & Li (2015)	By handling dynamic data transmission and execution together resource utilization can be improved effectively.
Li et al. (2016)	Develop a security framework for business cloud.
Arunarani, Manjula & Sugumaran (2017)	Develop a security framework for business cloud.
Poola, Ramamohanarao & Buyya (2016)	Considering the fluctuating prices of resources of data centers between regions and also the related data transfer costs to form a task placement strategy.
Rehani & Garg (2017)	Improve the proposed algorithm by considering other vital parameters like security and budget in a cloud environment.
Xie et al. (2017)	On heterogeneous systems reduce schedule length and minimize resource usage for parallel application.
Swain, Saini & Sahu (2020)	Extend the existing approach with if tasks are having other properties and if more than one VMs required for a task.
Duan et al. (2017)	In a real cloud scenario meet the requirement of resource-intensive applications.
Pietri et al. (2013)	Investigate the effects of costs of data transfer, workflow structures and provisioning delays on algorithms performance
Yassa et al. (2013)	How to achieve reliability and security in addition to energy consumption by improving the existing technique.
García, Espert & García (2014)	A decision making procedure is required for distributed SLA monitoring, for each type of violation the selection of corrective action, and a disaster recovery protocol.
Garg et al. (2014)	Aim to include multiplexing schemes when multiple VMs are combined on the same server to eliminate interference from the applications. Considering multi-core CPU architectures in addition to network and memory conflicts
Panda & Jana (2017)	To develop effective algorithms in the heterogeneous multi-cloud environment include parameters like performance, reliability and availability for its applicability.

### Cost aware scheduling

In Partitioned Balanced Time Scheduling (PBTS) algorithm workflow is executed in a set of homogeneous VMs through partitioned its execution therefore scheduling choices are prepared for each single billing period. For each partition or cycle, PBTS mainly recognizes the tasks set to process on the basis of considered resource capacity which particularly thinks about the overall cost. Its chief concern is to evaluate, the lowest number of calculating resources, for all scheduling partitions necessary in less than the user stated the deadline to execute the workflow. Formerly suggested by the same authors’ that the Balanced Time Scheduling (BTS) algorithm assessed the particular number of resources required during the partition to process the tasks. In conclusion, the tasks which are executed based on the schedule attained are distributed from processing BTS and the actual VMs.

A sub-workflow static hybrid method is adopted by the PBTS to manage the VM mapping tasks and each billing period statically scheduling tasks is a decent model of an algorithm. Each single scheduling cycle or partition enables the algorithm to have greater endurance to performance variability and gain benefits of the cloud flexibility by modifying the total numbers of VMs and observing tasks execution time. It as well makes use of runtime modification to address suspensions or delays on the tasks scheduled statically. The PBTS was undoubtedly considered for the coarse-grained billing periods or with finer-grained periods like 1 min or 1 h. PBTS might not be as effective as it would be challenging to allocate a huge number of tasks to every cycle because tasks are not likely to execute in less than a single cycle to make the scheduling useful Byun et al. (2011b), Byun et al. (2011a).

The Hybrid Cloud Optimized Cost scheduling (HCOC) algorithm is to accelerate the execution of work-flows, also decreasing costs when related to other approaches. It’s multi-objective working, can make available makespan as little as the user desires along with the cost awareness. In the cloud setup, the HCOC scheduling algorithm has the ability to decrease the execution costs with the increase of the workflow in the required execution time. HCOC scheduling algorithm offers well-organized scheduling in a hybrid cloud setup. As a result, HCOC makes it possible for the customer to control costs by regulating the preferred workflow execution time. Moreover, HCOC can be effortlessly modified to take care of budgets instead of the time limit, which makes it stress-free to use with different requests Bittencourt & Madeira (2011).

Workflow scheduling linked algorithms are chiefly dedicated to adjusting execution time or cost. Though, in cloud computing, workflow scheduling is adjacent to the pressures on the applications regarding in-built insecurity and unreliability. For that reason, must be considered trust service-oriented approaches. This algorithm provides balanced policies to empower consumers to equilibrate multiple desires, including time, trust, and cost. Furthermore, Trust service-oriented work-flow scheduling algorithm associates recommendation trust with direct trust by implements a trust metric (Tan et al., 2013).

A mathematical cost optimization scheduling model in which the cost of workflows is optimized on Iaas clouds under a deadline restraint presented by Malawski et al. (2012). It adopts a multi-cloud environment with heterogeneous VM instances, to share intermediary data files’ global storage facility is used where each supplier offers a restricted number of instances. This technique of putting together the scheduling problem as a mixed-integer program (MIP) recommends task placement and global optimization of data. In these two improved forms of the algorithm are offered, coarse-grained workflows for the tasks, with the execution time of 1 h, and to handle numerous small tasks fine-grained workflows with smaller than 1-h deadlines. This Cost optimization model has been specified in the CMPL and AMPL modeling language. Mixed-integer programming project to resolve large-scale scientific scheduling requirements on hybrid clouds, in which overall cost, with a deadline constraint, is the optimization objective.

A static, cost minimization, the deadline-constrained algorithm proposed by Rodriguez & Buyya (2014). Observe characteristics for instance heterogeneity of limitless computing resources and the elastic provisioning along with VM performance variation. Both Scheduling and resource provisioning are fused and demonstrated using a particle swarm optimization problem. Therefore a near-optimal schedule responsible for types and numbers of VMs that are used, besides their task to resource mapping, and the rental period is presented in the algorithm. The benefit of the algorithm is the global optimization procedure as it lets us put together high-grade schedules. The algorithm positively captured the boundless resource model; however, in the workflow computational directly increases with the types of VMs offered by the supplier and the number of tasks.

Mansouri & Javidi (2019) proposed Cost-based Job Scheduling (CJS) algorithm. The proposed algorithm makes use of data, network characteristics, and processing power for the job allocation procedure. Simulations are conducted by authors and matched results of CJS with other state of art algorithms. CJS technique is able to decrease the response time of scheduled jobs that might involve computing-intensive and data-intensive jobs.

### Load aware scheduling

Efficient load-based scheduling can comprise of numerous fine computational granularity jobs. When using numerous resources of a cloud frame, the runtime of these schedules may be smaller than the length of system overheads. Job clustering combines various small running jobs into a sole job as a runtime optimization practice hence the scheduling expenses are lessened and the whole runtime performance is enhanced. Nonetheless, prevailing job clustering approaches simply provide a coarse-grained technique that depends on an over-simplified workflow. Chen et al. (2015) observe the causes of dependency imbalance and runtime imbalance in task clustering and suggested quantitative metrics to assess the two imbalance problems and their seriousness. Additionally, recommend vertical and horizontal task balancing methods to explain the load balance problem when implementing job clustering for extensively used work-flows. The authors examine the connection between the performance of suggested task balancing methods and these metric standards. A trace-based simulation presents that the suggested methods when matched to a baseline execution considerably lessening the runtime of workflow applications.

Kumar, Gupta & Jana (2016) recommend a load-aware scheduling technique in cloud computing for workflow applications. The suggested algorithm is performed in two stages. In the first stage primacies of all the tasks are calculated then, in the second stage they choose VM and schedule tasks. After the execution of the present task, the complete load to be executed instantaneously, and this method is also taken into attention. The outcomes are compared with a variation of the proposed procedure and the standard heuristic scheduling called Heterogeneous Earliest Finish Time (HEFT). The results indicate that the HEFT technique extraordinarily shows the performance metrics such as an increase in average cloud utilization and reduction in makespan.

Balanced and file Reuse-Replication Scheduling (BaRRS) algorithm is suggested by Casas et al. (2017). According to the desirability schedule scientific application workflows in cloud environments. To balance system utilization BaRRS divides workflows through parallelization into numerous sub-workflows. Furthermore to enhance the total data files amount that is required to be relocated between tasks along with run-time it applies data reuse and replication scheduling techniques. Important application features assess by BaRRS such as file sizes, dependency patterns of scientific workflows, and task execution times for replication techniques to systems and adjusting current data reuse in the cloud. Also, BaRRS executes a balanced analysis centered on two optimization constraints to adopting the best solution one is a monetary cost and the other is the execution time of running workflows. The results show when BaRRS is compared with advanced scheduling techniques; it performs well. Investigations involved four up to date scientific workflows with altered data file sizes and dependency patterns. The proposed technique shows encouraging results and emphasized the most essential factors determining the execution of scientific applications in cloud computing.

As there are extensive resources and a lot of users addresses cloud computing load-balancing problems in consequence load-balancing practice is the major issue in the distributed cloud that many researchers considered that as an NP-hard problem so it could be the leading problem in the cloud. For that reason, previous researchers presented several heuristics algorithms for example Firefly Algorithm (FA) and the Imperialist Competitive Algorithm (ICA) to solve the load balancing problem. Though in explaining and solving load-balancing issues FA and ICA may perhaps get a satisfying result. An intelligent meta-heuristic algorithm based on ICA and FA combination presented by Kashikolaei et al. (2020) to get the improvements in load balancing, stability, makespan, planning speed and CPU time.

A load-balanced task scheduling algorithm presented by Panda & Jana (2019) focused on probability theory in the cloud environment. This proposed technique is presented through a time complexity of O(lm) to be an approximation algorithm, where m stands for the number of VMs, and l stands for the number of client tasks. Results of experimentation show that when we compared the suggested algorithm in four different performance measures with the existing algorithms, that is standard deviation of VM loads, zero loads, minimum load, and the maximum load it can extraordinarily balance the VMs load.

### Makespan aware scheduling

Niu, Ong & Nee (2012) improved a meta-heuristics, Intelligent Water Drops (IWD) algorithm, which is modified for resolving job-shop scheduling problems. To advance the original IWD algorithm, five arrangements are suggested and the upgraded algorithm is termed as Enhanced IWD algorithm (EIWD) algorithm. Investigational results prove that the modified algorithm has the ability to find better-quality solutions for the standard examples as compared to the prevailing makespan-based algorithms by using five strategies. The author advances the original IWD algorithm to escalate the solution quality along with the diversity of the solution space.

Consumers are charged according to their required QoS specifications and usage of resources in cloud computing. The workflow scheduling techniques which improve both cost and makespan are proposed by Zhu et al. (2015) for the cloud as a Multi-objective Optimization Problem (MOP). An Evolutionary Multi-objective Optimization (EMO)-based algorithm on Infrastructure as a Service (IaaS) stage is proposed to resolve this workflow scheduling problem. In this algorithm innovative patterns for problem-population initialization and specific encoding, genetic operators, and fitness evaluation are suggested. The schedules formed by our EMO-based algorithm show more constancy with most workflows having the IaaS computing and pricing models when experimentation is performed on randomly generated workflows and real-world workflows. The EMO-based algorithm can attain considerable results than existing up-to-date QoS optimization scheduling algorithms.

Poola et al. (2014) proposed a robust scheduling algorithm that schedules workflow jobs on heterogeneous Cloud resources by providing resource allocation policies that aim to reduce the makespan (total elapsed time) and the cost. The suggested resource allocation policies deliver fault-tolerant and robust scheduling while reducing makespan in experiments results.

Raju & Devarakonda (2019) proposed a technique, to the balanced cost of rendering service and make efficient utilization of resources. For this purpose along with Modified Ant Colony Optimization (MACO) along with Modified Fuzzy Clustering Means algorithm (MFCM) scheme is used as a result of using a cloud arrangement cost and makespan together with load balancing ability is deceased.

For hybrid clouds, Zhou et al. (2019) presented two efficient workflow scheduling techniques that both consider monetary cost and makespan. To reduce workflow's monetary cost under deadline constraint, Deadline-Constrained Cost Optimization for Hybrid clouds (DCOH) is proposed which is based on a single-objective workflow optimization method. Along with DCOH, for adjusting makespan and workflows monetary cost at the same time a multi-objective work-flow optimization method named Multi-objective Optimization for Hybrid clouds (MOH) is also proposed. Results of the simulation show that the DCOH technique can lessen the makespan related to the benchmark techniques.

### Security aware scheduling

High standard security service is progressively very vital for cloud workflow applications. Though, current scheduling approaches neglect the security needs of workflow applications for cloud systems. This resource competition might prominently affect the regulatory cost and computation time of both given tasks and related necessary security services. Zeng et al. present an immoveable dataset idea to solve this problem which makes the movement of certain datasets for the reason that of cost and security concerns and suggests an innovative scheduling model in the cloud. Security-Aware and Budget-Aware workflow scheduling approach (SABA), is proposed by Zeng, Veeravalli & Li (2015) to resolve the security problem which embraces a cost-effective distribution of tasks amongst accessible Cloud Service Providers (CSPs) within the market. To make available security services as well as a shorter makespan for customers.

A Security and Cost-aware Scheduling (SCAS) algorithm is proposed by Li et al. (2016) for heterogeneous scientific workflow applications in the cloud system. It focused on particle swarm optimization, on the meta-heuristic optimization approach, to decrease the total workflow execution cost the coding approach of which is developed with the risk rate constraints. The vast experimentation results demonstrate the practicality and efficiency of the algorithm as compared to the existing algorithms by using three real-world workflow applications along with CloudSim simulation.

The addition of security services to workflow applications in terms of execution time automatically causes outlays. In the cloud environment for workflow scheduling, the balance between attaining the preferred standard of security services and great computing performance is very daring. In the cloud, Arunarani, Manjula & Sugumaran (2017) presented security and cost-aware scheduling algorithm for heterogeneous tasks in scientific workflow applications. This technique combines Bat and Firefly algorithms based on hybrid optimization techniques. Extensive experimentation results show that the suggested algorithm leaves behind the existing algorithms as to reducing the over-all execution cost even though meets the risk rate constraints and deadline.

### Reliability aware scheduling

As a payment-based service cloud frameworks offer low-cost computing resources. These resources are provisioned with dynamism and flexibly accessible. In spot instances, cloud supplier’s established new pricing models specifically cost-effective that why users adopting cloud more and more for scientific resource applications. Though, when the market price goes beyond the consumer’s bid price, spot instances are ended. Consequently, the cloud environment is not faultless. Failures are unavoidable in such huge multipart distributed systems. In performance, cloud resources observe fluctuations which are also well-considered. These concerns make fault restraint a significant benchmark in scheduling techniques. For workflow scheduling, an adaptive, just-in-time scheduling algorithm is proposed by Poola, Ramamohanarao & Buyya (2016). This algorithm provides fault tolerance and reduces cost by carefully uses both on-demand and spot instances. The proposed scheduling algorithm also combines resources to further minimize cost and execution time. The proposed heuristics approach is fault-tolerant and effective is showed after performing extensive experiment simulations particularly under short deadlines, given that robust schedules with reduced cost and makespan.

Using the cloud resources up to their capability is advantageous in efficient job scheduling. Furthermore, it is significant to think about the reliability of computation resources while scheduling as the failure of tasks may be risky to both the user and the CSP in real-world circumstances. To present the failure types of a cloud environment Rehani & Garg (2017) presented a Monte Carlo Failure Estimation (MCFE) algorithm. Proposed algorithm by using the Monte Carlo simulation technique review Weibull distributed failures in the cloud framework, to find out the possible frequency of failures. To review the consistency and reliability of execution of tasks whereas allocating tasks in a scientific workflow application to VM also proposed Failure-Aware Resource Scheduling (FARS) algorithm. The authors compared the standard scheduling algorithm namely HEFT with the proposed MCFE and FARS. For simulation investigation randomly generated task graphs and task graphs for numerical real-world problems like Fast Fourier Transformation (FFT) and Gaussian Elimination (GE) were taken into consideration. The proposed algorithm performs better where reliability is a serious issue in real-world consequences.

In heterogeneous service-oriented systems, reliability is extensively recognized as a significant issue since the quality of service is interrupted by processor failure to cloud clients. To fulfill the application’s reliability constraint replication-based fault-tolerance is a frequent technique. In a heterogeneous service-oriented systems study of Xie et al. (2017) explains the challenge of reducing redundancy to fulfill reliability needs for a directed acyclic graph focused on parallel application. To meet the application’s reliability requirement authors put forward Enough Replication for Redundancy Minimization (ERRM) algorithm, and when to address with little time complexity in reliability requirement of the application. The authors present Heuristic Replication for Redundancy Minimization (HRRM). ERRM can generate minimum redundancy afterward HRRM proves at different scales, heterogeneity, and parallelism in randomly generated parallel applications. Furthermore, the lowest redundancy with a little computation time estimated when implements HRRM.

The possibility of failure of the resources rises with scale because cloud systems use data centers having a huge amount of computational resources. To study the reliability issue it is vital to keep in view the failure of the resources, for application utilization in the cloud. Failures are the reason for the absence of services, which interrupt the reliability of the cloud environment. Swain, Saini & Sahu (2020) analyze, design, and provide an explanation of two special problems which are tasks with corresponding execution time and with usual deadline with the same failure ratio on the machines and present in the cloud the reliability aware scheduling with deadlines. Authors propose two-phase heuristic techniques, task orders and task mapping to machines. Results of various task ordering and task mapping approaches are estimated through real and synthetic traces simulation. Results of the simulation show besides extensive task dropping the initial due date task ordering and task mapping to the best reliable machine performs well in general situations and task replication and repetition further progress the performance of the heuristics.

### Energy aware scheduling

How to lessen energy consumption and keeping up with high computation capacity has come to be a well-timed and vital challenge with the fast growth of cloud computing. Existing VMs scheduling techniques have largely concentrated on decreasing power consumption by boosting the legacy “bin-packing” algorithm and improving the cluster resource utilization. Nonetheless, various resource-intensive workflow applications in practical scenarios running on VMs have important effects on energy consumption and system performance. Moreover, the energy efficiency of scheduling algorithms can expressively slow down when instant peak loads cause a scheduling error. A prediction model based on an improved ant colony algorithm focus on fractal mathematics and a scheduler. Duan et al. (2017) presented a new scheduling technique called pre Ant Colony. The model finds out how to initiate the execution of the schedule by the integrity of load trend prediction and whether the scheduler is accountable for resource scheduling while reducing energy consumption under the basis of assuring the QoS. By using real workload traces through extensive simulation experiments, the performance outcomes show that the given approach displays excellent resource utilization and energy efficiency. Furthermore, in a heterogeneous computing environment for resource-intensive applications this technique suggests an efficient dynamic capacity provisioning example and while scheduling is generated through instantaneous peak loads can decrease the consumption of energy and system resources.

Discussing the issue of undergoing energy constraints alongside either deadline or budget constraints. For scientific workflow ensembles, Pietri et al. (2013) focused on the providing of resources. For task scheduling and resource provisioning, two energy-aware algorithms are presented by the authors. Utilizing synthetic data centered on real workflow application parameters extensive experimental simulation results demonstrate that the suggested algorithms reduce energy consumption and meet constraints without giving in the total workflows completed in an ensemble.

The hybrid PSO algorithm to optimize the scheduling performance presented by Yassa et al. (2013) to reduce energy consumption methodology is focused on the Dynamic Voltage and Frequency Scaling (DVFS) approach. This approach allows processors by given up clock frequencies to function in various voltage supply levels. Between the energy and quality of schedules, these multiple voltages involves settlement. On synthetic and real-world scientific applications simulations are performed and results focus the robust performance of the presented approach.

### Service level agreement aware scheduling

The manual management of the complexity and scope of the cloud becomes a challenging problem as the system required more employees, expertise, and resources. Widespread infrastructure management meanwhile supporting numerous dynamic requirements of consumers are well handled by SLA based autonomic cloud solutions. Cloud algorithm that able to complete the resource lifecycle introduces by García, Espert & García (2014) is termed Cloudcompaas, an SLA-aware PaaS cloud algorithm. Cloudcompaas allows providers of clouds with a generic SLA model. Designed with specific requirements of cloud computing, this algorithm features an extension of the SLA requirement WS-Agreement to agree with closer to end-user perception and higher-level metrics. Furthermore, Cloudcompaas offers a framework for common applications of cloud computing that might be utilizing cloud infrastructures elasticity features with dynamism modified to precise the QoS violations. The efficiency of this strategy is shown by the results of a simulation that addresses numerous realistic workload profiles, in which Cloudcompaas under extremely heterogeneous utilization patterns managed maximum efficiency and minimum cost.

Web applications use datacenter resources inefficiently shows that previous work either concentrating on a single type of SLAs or resource usage protocols of applications. The resource allocation issue in a datacenter tackled by Garg et al. (2014) runs various types of workloads of application, mainly transactional and non-interactive applications. The authors presented an admission control and scheduling procedure that not only satisfies QoS requests of users as stated in SLAs but also increases resource utilization and throughput. Experimental studies show it is vital to be aware of various types of SLAs besides applicable penalties and the mix of workloads for enhancing utilization of datacenters and resource provisioning. The proposed technique offers substantial decreases in SLA violations and improvement over static server consolidation.

SLA-MCT and SLA-Min-Min, two SLA-based job scheduling algorithms were presented by Panda & Jana (2017) for a heterogeneous multi-cloud environment. Single-phase task scheduling is presented in SLA-MCT; however two-phase task scheduling is presented in SLA-Min-Min. The suggested algorithms include the violation cost of SLA for the ineffective completion of the service as demanded by users and the gain cost of SLA for the effective end of the service as decided by the user. Deciding by the customers covers three levels of SLA. Moreover, the authors, using synthetic and benchmark datasets, simulate the proposed algorithms. The extensive simulation, in terms of four performance metrics that is, penalty cost, makespan, the gain of the services, and average cloud utilization is conducted and outcomes of suggested SLA-MCT matched with three single-phase and the outcomes of the suggested SLA-Min-Min are matched with two-phase existing task scheduling algorithms. The results noticeably display that in contrast with other algorithms the given algorithms suitably balance between gain cost and makespan of the services.

## Conclusion

### Discussion: open challenges

On pay per use basis, computing infrastructures are effortlessly applied by cloud computing and a lot of advancement has been done. However, we need to address many challenges and issues that are in this field. We have recognized that many open challenges are still pending in this area according to existing research in cloud scheduling. After observing various Scheduling techniques of the past decade mention in Table 1, answers to many questions are unresolved, doubtful, or neglected for example how does consumption of power in terms of the SLA affect user satisfaction? How to find fault tolerance and VM migration instantaneously in the cloud? How the relationship between performance and network load observe the arrival of tasks in the form of batches? How to achieve reliability and security in addition to energy consumption? How to reduce computational time when the complexity of instances increases? How to handle load/store bandwidth issues when workflow applications are data-intensive? How to change the resource capacity with dynamism according to the level of resources and workflow?

From the existing literature following issues and open challenges have been identified.

### Resource scheduling

In a cloud environment, the main challenge of resource scheduling comprises resource heterogeneity and incredibility that are not explained with traditional resource controlling procedures. Hence, this is necessary by considering these properties of the cloud, make efficient cloud applications and cloud services. The purpose of resource scheduling is to distribute the right workloads to suitable resources at the right time, therefore the applications in cloud computing can efficiently make use of the resources. To maintain a necessary level of QoS, the number of resources must be reduced for a workload, or decrease completion time of workload. New algorithms need to be developed to address this issue.

### Reliability

A necessary quantity of resources is provisioned by CSP to achieve the QoS requirements of cloud service. One of the utmost thought-provoking concerns is reliability because based upon the performance and cost now service provider offers the facility to the end-user. To provide a competitive service a real complication which service provider encountered is how to fulfill the vital need of users like scalability, reliability, and additional major QoS parameters. The scheduling failure rate will increase with the low reliability of the cloud, as a result, the cost and makespan will both increase. In order to enable reliable resource scheduling, two important matters need to be considered the first one is according to the resources information how to implement reliable scheduling and the second one is how to evaluate the reliability of the resources. Thus, in future research reliability is considered as a challenge in cloud resource scheduling.

### Energy management

To minimize the effect on the environment worldwide government’s organizations, and individuals, have shown an extraordinary interest to lessen carbon footprints. The progress in energy efficiency is one of the chief concerns in cloud computing. It has been assessed recently that the total operational expenditure of data centers, the price of refrigeration and power is very high. For that reason to reduce energy consumption infrastructure providers are under huge pressure. Not only to meet environmental standards and government rules but also to reduce cost of energy in data centers. In the past decade, few algorithms have been developed that are focused on the energy used up by the task execution. They focused on a combination of challenging scheduling objectives as they attempt to find a solution concerning performance, cost, and energy consumption. To reduce power consumption turning off unoccupied systems, server consolidation and energy-efficient task scheduling are considered. To achieve a reasonable trade-off between energy savings and performance of the application is an important issue in existing techniques. Many researchers until now are struggling to determine an innovative balanced solution between the availability of end-user requests and energy efficiency.

### Data security

In cloud computing, data security is another open challenge. Generally, in data centers, the physical data security system cannot be accessed by CSPs. To attain complete data security CSPs need to be dependent on the infrastructure provider. This is risky to form trust measures at every cloud architectural layer. By using secure VM observers the virtualization platform must be trustworthy. Concerning QoS complications, researchers have paid more attention to parameters like cost and makespan and neglect security. In scheduling workflows of clouds, data security and privacy protection are crucial problems to be solved. For that reason, focusing on security challenges must be of prime importance, which subsequently to plan an effective solution, improve the amount of trust-worthiness of resources, and enhance the flexibility and robustness of scheduling methods. Therefore in cloud computing how to secure and execute private data safely may be a problem worth reviewing. Presently cloud applications are very data-intensive, with a huge quantity of data supported by the cloud atmosphere so, the data transfer and resource management between computing resources and storage are the main restrictions in the cloud. It is necessary to find a valuable approach to look out for the big data required by the workflows.

### Service level agreements

SLAs in terms of SLA autonomic cloud infrastructures is required to carry out the cloud quality service requirements demanded by cloud customer and also to reduce interaction between the cloud consumer and the computing environment. SLA is planned and SLA violations are identified on a regular basis based on these QoS requirements. Thus, to avoid or reduce SLA violations this is required to dynamically provision appropriate quantity of resources by cloud service provider. Also, an effective strategy is required in advance to detect SLA violations to avoid performance degradation.

By evaluating numerous highly developed scheduling techniques from 2011 to 2020, it has been noticed that a single algorithm cannot fix or cover all the problems for example if one algorithm or scheduling technique focused on parameters like time, cost, and security whereas other technique completely neglect that parameters and concentrate on different parameters such as energy consumption, response time, availability, load balancing, etc.

In the cloud computing environment, several research limitations are still unresolved. In the past to develop the vital performance indicator features, several resource managing techniques have been suggested but still not beneficial to combat the challenges, because of certain complications such as the complicated nature of applications, random requirements of end-users regarding load balancing and storage, energy consumption, unpredictable cost virtualization, reliability, makespan, and security, etc. that have not been broadly and systematically studied so far also with no advance research direction.

### Future research guidelines

Future research should center on resolving the limitations already faced by researchers along with providing innovation to improve the cloud horizon. In the future following attempts can be made to deal with the limitation of existing algorithms;

- Development of efficient protocols and algorithms that run under resource constraints without compromising the high level of security that is essential to protect the private data.
- To reduce failure likelihood in reliability machine learning techniques could be used as a service. In a cloud computing environment, more compelling machine learning approaches and workload prediction techniques are required to be established as it is challenging to foresee the impending.
- Cloud is a business model. In the execution of applications, the main concern of users like security, reliability, and SLA maintenance should be focused on and develop an efficient and safe framework for the business cloud.
- Comprehensive research is required for resource scheduling based on energy because green computing is a demanding issue for the cloud in the coming days. There are specific fundamental mechanisms like task migration, VM migration, monitor the memory utilization, and define a threshold value, etc. that must be controlled in an organized manner.
- Network bandwidth parameter is usually don’t consider by most of the existing scheduling techniques that may lead to communication delay problem, data loss, and network failure so, in future, one must think through this before developing an algorithm to work out these issues.
- SLA violations need to be identified at the time of resource provisioning. For the selection of corrective action for each type of violation, disaster recovery protocol, and distributed SLA monitoring a decision-making system is required.
- New scheduling algorithms can be proposed, consisting of heuristic and meta-heuristic techniques along with a cost-effective model to achieve reliability and security in addition to energy consumption and satisfying the need for end-user.
- To increase the efficiency of the entire cloud computing system task migration and failure management should be added in future research since there are insufficient scheduling techniques that pay attention to task migration and failure management
- To increase efficiency and decrease the amount of data transported to the cloud for analysis processing, and storage fog computing can be used. It may also be used to deal with the drawbacks of traditional elastic cloud like security and compliance. Fog computing provides a high level of decentralization and heterogeneity.

Slowly but surely, more businesses will store their data in the cloud and will bond with service providers to execute data analytics using the cloud environment. Shortly, companies will have no other option than to store their data in the cloud. The business struggle will largely depend on data safety and the ability to access and share data. Organizations are probably to become ever more codependent. Future generation cloud should be all set for nano-computing and quantum computing types of nontraditional architectures. These technologies with far-reaching impact will stimulate future applications. Provide high performance, fast processing, and plentiful storage with low power consumption.

## Additional Information and Declarations

### Data Availability

The following information was supplied regarding data availability: This is a literature review. No data or code are available.
